# Spontaneous Triceps Haematoma Presenting With Ulnar Neuropathy: A Report of a Rare Case

**DOI:** 10.7759/cureus.115764

**Published:** 2026-09-04

**Authors:** Lahiru Mudalige, Bharath Sundaramoorthy, Muthusankar Sudalaimuthu, Melissa Benn, Conal Horgan, Deepak Verma, Suresh Pillai

**Affiliations:** 1 Emergency Medicine, Swansea Bay University Health Board, Swansea, GBR; 2 Faculty of Medicine, Health and Life Science, Swansea University, Swansea, GBR

**Keywords:** antiplatelet drugs, antithrombotic therapy, spontaneous intramuscular haematoma, triceps muscle, ulnar nerve neuropathy

## Abstract

Spontaneous intramuscular haematoma of the triceps muscle has the potential to pose significant diagnostic and management challenges. We report the case of a 42-year-old man presenting with acute non-traumatic right upper limb pain and a palpable posteromedial mass. He was receiving antiplatelet therapy for a previous transient ischemic attack. Examination revealed a tender mass on his triceps region with mild ulnar sensory deficit. Imaging demonstrated a well-defined cystic lesion; however, malignancy could not be excluded. Ultrasound-guided aspiration subsequently confirmed a haemorrhagic collection consistent with a haematoma. The patient was managed conservatively with planned radiological follow-up. This case highlights the importance of considering spontaneous haematoma in patients presenting with atraumatic soft tissue masses, particularly those on antithrombotic therapy. Careful imaging, histological evaluation when indicated, and appropriate follow-up are critical to exclude malignancy and guide management.

## Introduction

Spontaneous intramuscular haematomas (SMH) are defined as bleeding within muscle compartments in the absence of significant trauma or iatrogenic injury. Although uncommon, they represent a clinically important entity due to their potential to mimic more serious pathologies and, occasionally, lead to significant morbidity. These lesions are most frequently reported in the iliopsoas, rectus sheath, and gluteal muscles. Involvement of the upper limb, particularly the triceps muscle, is exceedingly rare [1,2].

The incidence of spontaneous muscle haematomas is associated with advanced age, anticoagulation therapy, coagulopathies, or minor unrecognised trauma. When compared to antiplatelets, there is a higher association between anticoagulants and the incidence of SMH [3]. However, such haematomas may also occur in patients without overt coagulation abnormalities, posing diagnostic challenges. 

Clinically, SMH can present as painful soft tissue masses and may be associated with neurological deficits due to compression of adjacent neurovascular structures. Importantly, their presentation can closely resemble soft tissue tumours, particularly when imaging demonstrates a well-defined mass with internal complexity [4]. This overlap creates a diagnostic dilemma, as intramuscular bleeding may occasionally obscure or coexist with an underlying malignancy, necessitating careful evaluation and follow-up. 

Given their rarity in the upper limb and the potential for misdiagnosis, spontaneous triceps haematomas remain underreported. Awareness of this entity is crucial for early recognition and appropriate management, especially in patients receiving antithrombotic therapy. In this report, we describe a case of a spontaneous triceps haematoma presenting as a suspicious soft tissue mass, highlighting the diagnostic challenges and the importance of excluding underlying neoplastic pathology.

## Case presentation

A 42-year-old man presented to the emergency department (ED) with a 1-day history of acute-onset right upper arm pain. He denied any preceding trauma or any recent strenuous physical activity. Functionally, he was able to perform light activities. The patient had a past medical history of essential hypertension, hypercholesterolemia, metabolic syndrome and a previous transient ischemic attack. His regular medications were amlodipine, atorvastatin, candesartan and clopidogrel. He denied the use of anabolic steroids, anticoagulants or any other relevant medications. Of note, the patient reported a similar episode involving the same site approximately six months earlier. At that time, the patient experienced swelling without any associated pain or neurological deficit. The patient underwent assessment in a private clinic, where magnetic resonance imaging (MRI) suggested a haematoma. Conservative management was advised. The swelling resolved spontaneously and he remained asymptomatic until the current presentation to ED.

On physical examination, the patient was conscious, oriented and clinically stable. His vital signs were as follows: blood pressure of 108/74 mmHg, pulse rate of 92 beats/minute, temperature of 37.0 °C, oxygen saturation of 98% on room air. A firm tender mass was palpable on the posteromedial aspect of the right upper arm. There was no overlying erythema, rash, increased localised temperature or any other skin abnormality. Neurological examination revealed mildly reduced sensation in the right ulnar nerve distribution distally. Sensation in all other dermatomes and a comprehensive motor examination were normal. Radial and ulnar pulses did not show any deficit as compared to the other side.

Laboratory investigation demonstrated a white blood cell count of 16.5 10^9/ L with neutrophils of 14.3 10^9/ L and a platelet count of 308*10^3/µL. The C-reactive protein level was mildly elevated at 21 mg/L. Renal function tests, liver function tests, bone profile and coagulation profile were within normal limits. Bedside ultrasound revealed a predominantly cystic mass with fine internal septations. The patient was referred to the plastic surgery team in view of the swelling and upper limb neurology.

Reviewing the patient, the plastic surgery team requested an ultrasound of the right axilla that showed features suggestive of a triceps haematoma (Figure 1). In the context of the current presentation, although the lesion was considered most likely to represent a haematoma, the plastics team initiated a soft-tissue lump diagnostic MDT (multidisciplinary team) referral, and the patient was later discussed in a sarcoma MDT. The MDT recommended exclusion of a sarcoma that had bled. Therefore, the patient had a CT thorax, abdomen and pelvis on the following day to rule out an underlying primary malignancy and to assess whether the soft tissue lesion could represent metastatic disease. The CT scan showed no evidence of malignancy; however, there were features suggestive of low-volume lymphadenopathy on the left side of the neck, for which an ultrasound (US) of the neck was performed. The US of the neck suggested no pathological cervical lymphadenopathy. Subsequently, an MRI scan of the right upper arm was performed, as recommended by the radiology team, which showed a haematoma; however, they were not definitely able to exclude an underlying small sarcoma that could have bled (Figure 2). Subsequently, it was decided to undertake a US-guided aspiration of the lesion to exclude the possibility of a sarcoma (Figure 3). Approximately 10 mL of haemorrhagic fluid was aspirated and was sent for histology and cytology. The histology showed inflammatory cells and haemosiderin deposition that was suggestive of origin from a haematoma, with no evidence of malignancy. The cytology report showed white blood cells, degenerate cells and debris with no evidence of malignancy. Therefore, the sarcoma MDT recommended a repeat MRI at three months to ensure the resolution of the haematoma.

**Figure 1:**
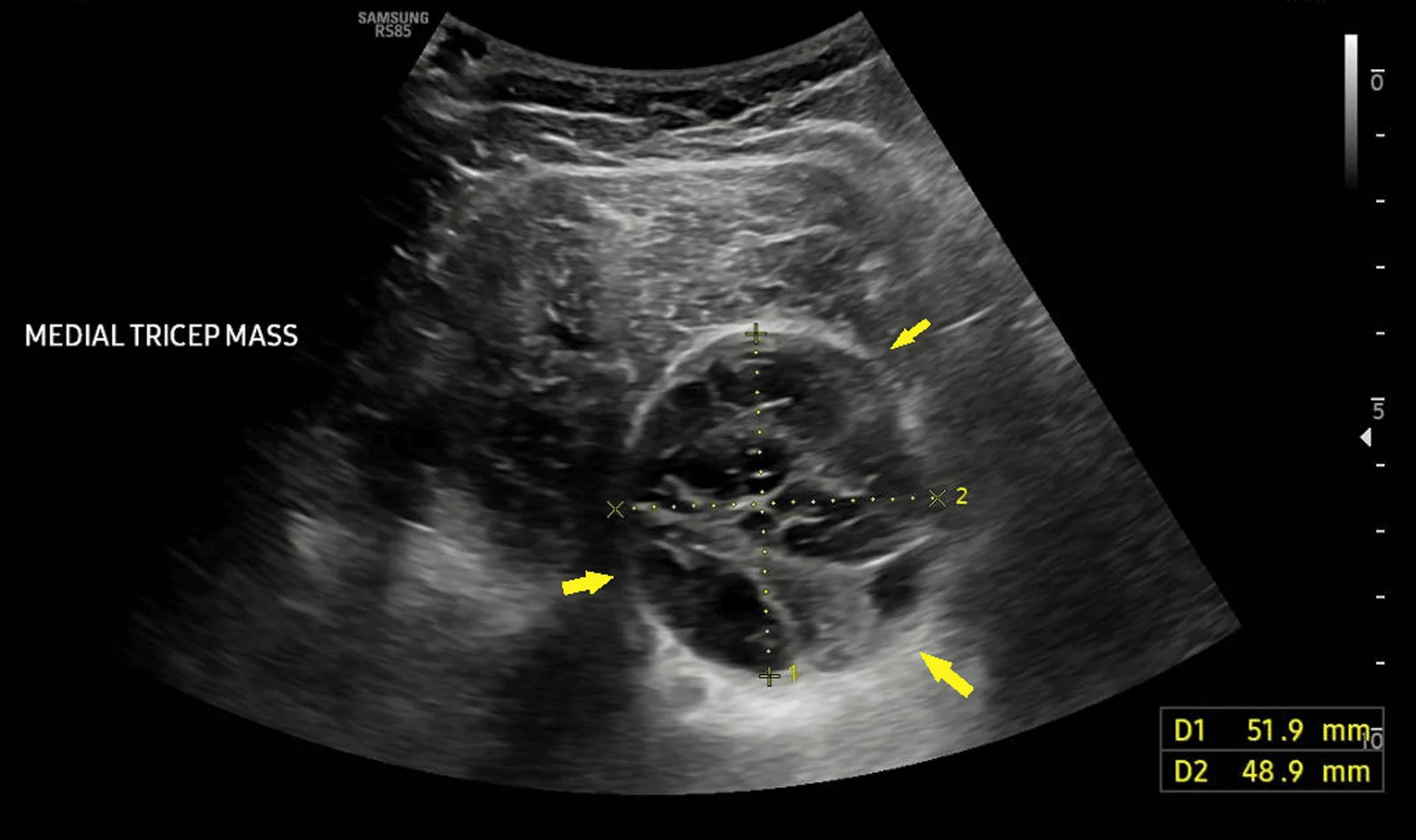
Ultrasound image of the localised collection centred on the medial proximal right triceps muscle with associated distortion of the adjacent lateral triceps; this could represent a haematoma

**Figure 2:**
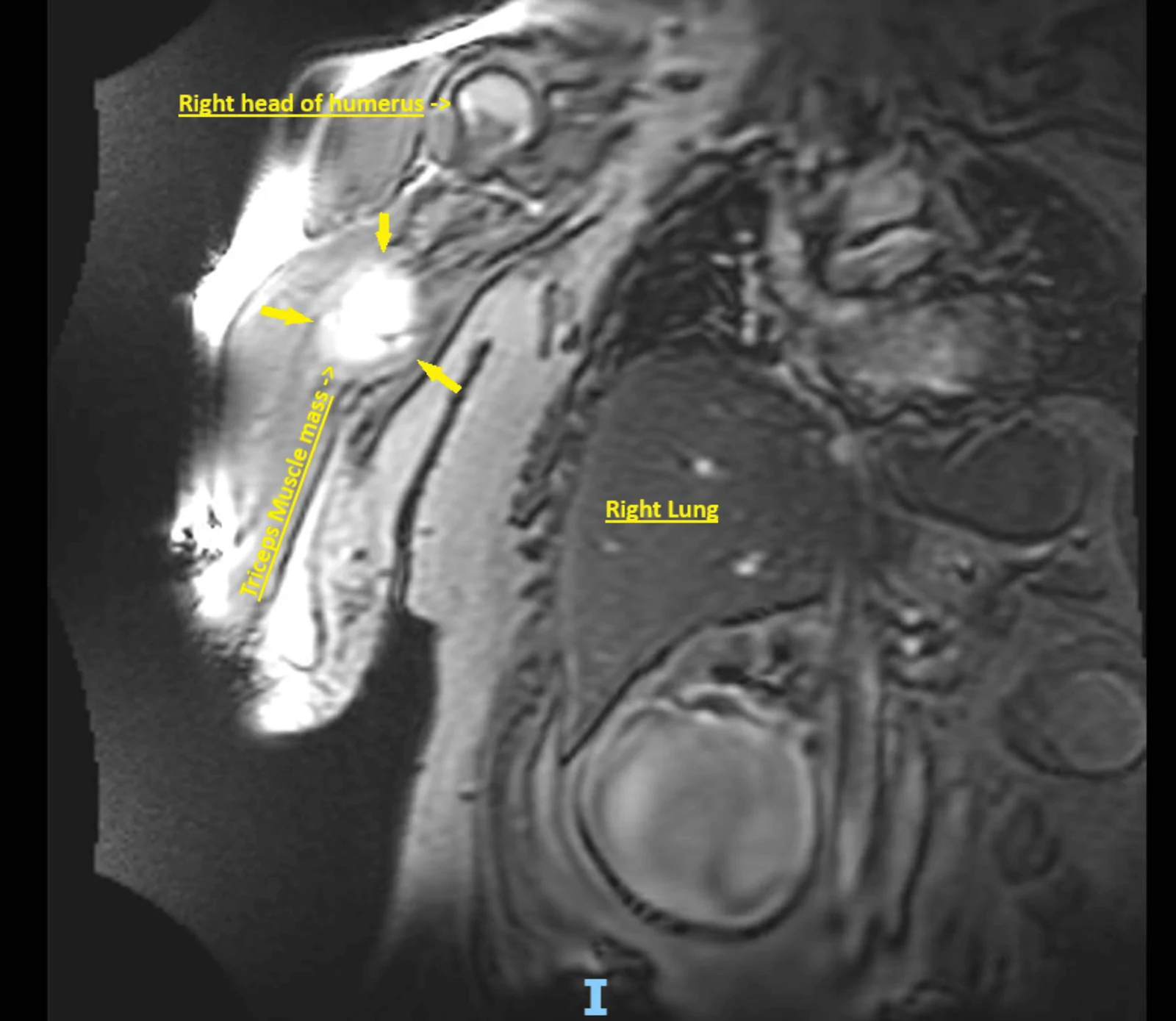
MRI scan showing a 6.5 x 6 x 5.5 cm mass identified in the posteromedial side of the arm The lesion is wedged between the posterior and anterior compartments of the arm. Most of the neurovascular bundle lies anterior to this lesion. The lesion has a fluid signal on fluid-sensitive sequences with a well-defined capsule.

**Figure 3:**
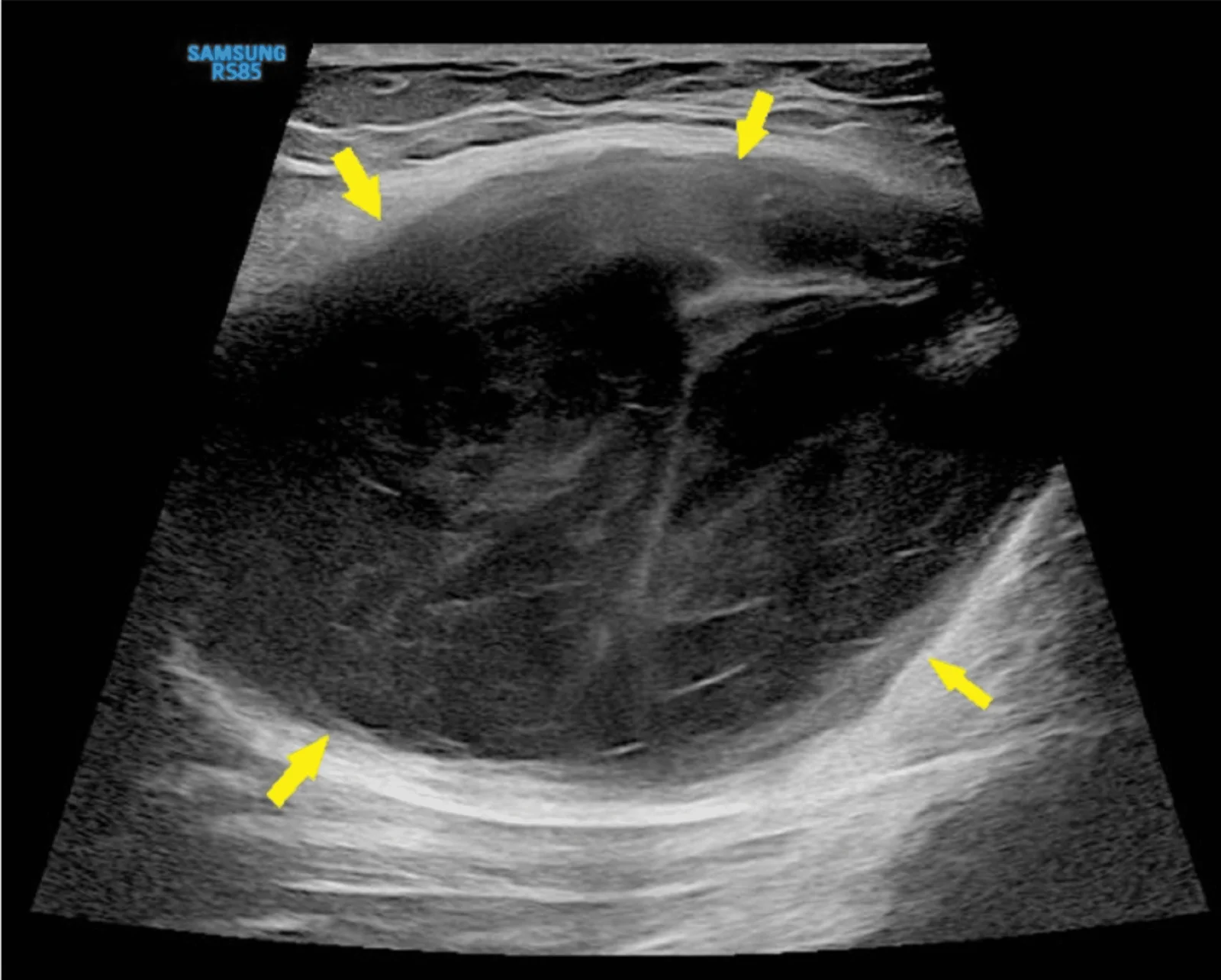
Ultrasound image of the lesion showing a predominantly cystic mass with fine central septa and a thick eccentric ring of partially neovascularised soft tissue, the appearance favouring that of an encapsulated haematoma

## Discussion

SMH are an uncommon clinical entity that can present diagnostic and therapeutic challenges, particularly when arising in atypical locations such as the triceps muscle.

Notably, this case occurred in a patient receiving antiplatelet therapy rather than anticoagulation, demonstrating that spontaneous intramuscular haematomas can arise outside traditional high-risk groups. While anticoagulants remain the most well-recognised predisposing factor, alternative mechanisms, such as minor unrecognised muscle injury, increased vascular fragility or underlying microangiopathic changes, may contribute [2]. This highlights the need to maintain clinical suspicion even in patients without evident coagulation abnormalities.

SMHs most frequently affect the iliopsoas, rectus sheath, and lower limb musculature [5]. Consequently, spontaneous triceps haematomas are rarely reported and may not be readily recognised, increasing the likelihood of misdiagnosis. In the present case, the absence of trauma and the presence of a well-defined mass raised diagnostic uncertainty, particularly in distinguishing a haematoma from a soft tissue neoplasm. This diagnostic challenge is well-recognised in the literature, where intramuscular haematomas can mimic malignant tumours, both clinically and radiologically [2,4].

Radiological assessment plays a central role in diagnosis. US is useful as an initial modality and for serial monitoring, while computed tomography (CT) and magnetic resonance imaging (MRI) offer greater diagnostic accuracy. MRI is valuable in differentiating haematomas from soft tissue tumours, although overlap in imaging characteristics may persist, especially in organising large lesions [3]. In the present case, the presence of a well-defined encapsulated mass with internal fluid signal intensity and surrounding oedema on MRI raised concern for underlying malignancy, particularly haemorrhage within a neoplastic lesion. This overlap necessitated careful diagnostic evaluation, including image-guided aspiration or biopsy, to exclude sarcoma.

Another important consideration is the potential for neurovascular compromise. Compression neuropathy is a recognised but rare complication of intramuscular haematomas. Depending on the size and location of the haematoma, symptoms may range from mild sensory deficits to severe motor impairment or compartment syndrome. In the upper limb, even moderate increases in compartmental pressure can affect neurovascular structures due to confined anatomical spaces, which likely explains the mild ulnar sensory deficit observed in this patient [5,6-9].

Management in such cases remains controversial, particularly regarding the role and timing of surgical intervention. In hemodynamically stable patients without progressive neurological deficits, conservative management, including analgesia, correction of coagulopathy, and close observation, is generally recommended. Current literature suggests that up to 85% of hematoma-related neuropathies improve without surgical intervention [9]. However, surgical or interventional radiology management may be required in specific situations, such as active bleeding, increasing size, or significant compression of neurovascular structures [2,7].

In such situations, early decompression appears to be associated with improved outcomes by reversing ischemia and decreasing the pressure effect. Notably, even small-volume drainage has been reported to provide symptomatic benefit by reducing compartmental pressure [7]. Haematomas evacuated within 48 hours have been shown to result in complete neurological recovery, whereas delayed intervention beyond this period reduces the success rate to approximately 50%, with a higher likelihood of persistent deficits [9].

Consequently, while a conservative approach may be appropriate in stable patients, delayed treatment in the presence of evolving neurological compromise may lead to irreversible damage [2,9]. Nonetheless, the lack of high-quality data and the rarity of such cases mean that there is no consensus on the optimal management strategy [8].

In the present case, ultrasound-guided aspiration was performed primarily for diagnostic clarification rather than therapeutic decompression. The relatively mild neurological involvement and absence of progressive deficits supported a conservative management approach with close follow-up. This strategy highlights the importance of individualised decision-making, taking into account clinical stability, the severity of neurological impairment and diagnostic uncertainty.

Overall, this case emphasises several important points: first, SMHs should be considered in the differential diagnosis of atraumatic soft tissue masses; second, distinguishing a spontaneous hematoma from an underlying soft tissue malignancy remains a clinical challenge because soft tissue sarcomas can bleed spontaneously; and third, early recognition of neurological involvement is crucial to guide management. A structured approach incorporating imaging, clinical monitoring, and timely intervention is essential to optimise outcomes and prevent long-term complications.

## Conclusions

This case highlights the importance of considering spontaneous haematoma in the differential diagnosis of atraumatic soft tissue masses in patients on antithrombotic therapy. A thorough evaluation, including imaging and tissue sampling, is essential to exclude malignancy and guide appropriate management.
